# Application of Acoustic Emission on the Characterization of Fracture in Textile Reinforced Cement Laminates

**DOI:** 10.1155/2014/178020

**Published:** 2014-01-30

**Authors:** J. Blom, J. Wastiels, D. G. Aggelis

**Affiliations:** Department of Mechanics of Materials and Constructions, Vrije Universiteit Brussel, Pleinlaan 2, 1050 Brussels, Belgium

## Abstract

This work studies the acoustic emission (AE) behavior of textile reinforced cementitious (TRC) composites under flexural loading. The main objective is to link specific AE parameters to the fracture mechanisms that are successively dominating the failure of this laminated material. At relatively low load, fracture is initiated by matrix cracking while, at the moment of peak load and thereafter, the fiber pull-out stage is reached. Stress modeling of the material under bending reveals that initiation of shear phenomena can also be activated depending on the shape (curvature) of the plate specimens. Preliminary results show that AE waveform parameters like frequency and energy are changing during loading, following the shift of fracturing mechanisms. Additionally, the AE behavior of specimens with different curvature is very indicative of the stress mode confirming the results of modeling. Moreover, AE source location shows the extent of the fracture process zone and its development in relation to the load. It is seen that AE monitoring yields valuable real time information on the fracture of the material and at the same time supplies valuable feedback to the stress modeling.

## 1. Introduction to Acoustic Emission

**Figure 1:**
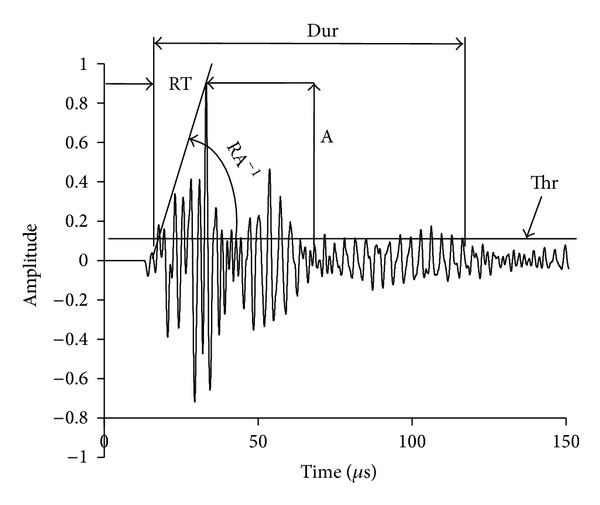
Typical AE waveform.

Acoustic emission (AE) is the result of irreversible processes in a material: mainly crack propagation, material failure, and changes in the microstructure [1]. Any fracture incident releases energy in the form of elastic waves and is captured by piezoelectric sensors on the surface of the material. These waves are recorded as electric waveforms. The number of acquired signals is connected to the number of active sources, while the location of the source events can be calculated by the time delay between the recording of each signal by sensors at different positions [2–5]. Some important parameters of the waveforms are shown in Figure 1. Among others, amplitude (A) is the voltage of the maximum peak and is connected to the intensity of the cracking event. Rise time (RT) is the delay between the onset and the maximum peak, and RA is the inverse of the rise angle (RT/A) and is connected to the mode of fracture [1, 6]. Additionally, frequency is checked by the “average frequency” AF, defined as the number of threshold crossings over the waveform duration. Duration (Dur) is the time between the first and last threshold (Thr) crossings. The area under the rectified signal envelope is another measure of the waveform's intensity and is called MARSE (measured area under the rectified signal envelope) or simply “energy” [6]. The threshold is selected by the user and should be high enough to exclude recording of ambient noise but at the same time sensitive enough to allow recording of even small amplitude relevant waveforms.

In the present paper a case of AE monitoring during fracture of a novel engineering material is presented. The aim is to demonstrate the capacity of AE to characterize the damage development and provide important information in the modeling of materials by indicating the dominant fracture mode under specific loading patterns.

## 2. Monitoring of Textile Reinforced Mortar under Bending

With the increasing prevalence of composite materials, the structural and cost advantages of textile reinforced cements (TRC) are well established [7]. The mechanical properties and production costs of TRC are dependent on physical, chemical, and production properties and methods. TRCs differ from fiber reinforced cements (FRC) primarily in their fiber structure, which is continuous. In combination with a usually higher fiber volume fraction (above 5%), this leads to an increased tensile strength and ductility. Within TRC materials, the development of inorganic phosphate cement (IPC) composites at the Vrije Universiteit Brussel (VUB) has resulted in a novel material [8]. The cementitious material shows a neutral pH after hardening; thus reinforcing with E-glass fibers is possible without fiber protection. By using a high volume fraction of E-glass fiber a relatively cheap strain hardening textile reinforced cementitious composite can be produced. When designing construction elements it is important to predict the bending behaviour of this composite. In this work the behaviour of straight and curved beams is examined in bending. The hyperelasticity “Marlow” material model in Abaqus was calibrated using experimental data from tension and compression tests. The calibrated model was used to study the bending behaviour of a beam, without taking into account the effect shear. The first objective of this research is to evaluate the influence of geometrical properties on the failure mechanism of an IPC TRC beam. The validation of the FEM analysis and limitations due to the introduction of a more complex failure mechanism is the second goal. Acoustic emission procedures are employed to measure the change in the failure mechanism and Abaqus is used to perform the structural analysis.

### 2.1. TRC in Tension and Compression

According to the ACK theory [9], three distinct stages can be detected in the stress-strain curve of a unidirectionally reinforced brittle matrix composite. In the first stage the material behaves linearly elastically and a perfect elastic bond between matrix and fibers is assumed. Since the failure strain of the matrix is lower than that of the fibers, the matrix will crack. If the fiber volume fraction is higher than the critical fiber volume fraction, the fibers will be able to sustain the cracking load, and multiple cracking will occur. In the third “post cracking” stage the matrix is fully cracked, and the fibers will carry any additional load until they fail.

### 2.2. TRC Beam in Bending

A hyperelasticity “Marlow” material model was used for bending analysis in Abaqus [10], because it enables the user to define a different behaviour in compression and tension. The tensile stress-strain data was taken by averaging the experimental data obtained from 10 samples. The compressive behaviour is assumed linear with a composite Young's modulus of 17.07 GPa, until collapse at 80 MPa. Results of modeling for a straight and a curved beam will be presented and discussed along with the experimental ones.

## 3. Experimental Part

### 3.1. Materials and Loading

The matrix is a mixture of a calcium silicate powder and a phosphoric acid based solution of metal oxides. The liquid to powder mass ratio is 1/0.8. Mixing is performed using a Heidolph RZR 2102 overhead mixer. First the liquid and the powder are mixed at 250 rpm until the powder is mixed into the fluid, after which the speed is increased to 2000 rpm. E-glass chopped glass fiber mats with a surface density of 300 g/m² (Owens Corning M705-300) are used as reinforcement. All IPC 8-layer laminates are made by hand lay-up with an average matrix consumption of 800 g/m² for each layer, which results in an average fiber volume fraction (*V*_*f*_) of 20%. Laminates are cured under ambient conditions for 24 hours. Postcuring is performed at 60°C for 24 hours while both sides are covered with plastic sheet to prevent early evaporation of water. Two sets of laminates are prepared: one flat and one curved with a base length of 282 mm and a vertex height of 138 mm. Basic parameters of the specimens are shown in Table 1.

### 3.2. Tensile Test

The stress-strain curve data was generated using flat coupons of the material on a universal testing machine (INSTRON 5885H) with a capacity of 100 kN. The rate of cross-head displacement was set to 1 mm/min. The strain was measured with a double clip extensometer. The stiffness in the first (*E*_*c*_1__ = 16 GPa) and the third stage (*E*_*c*_3__ = 4 GPa) is obtained from the experimental data by determination of the slope of the graphs.

### 3.3. Bending Test

**Figure 2:**
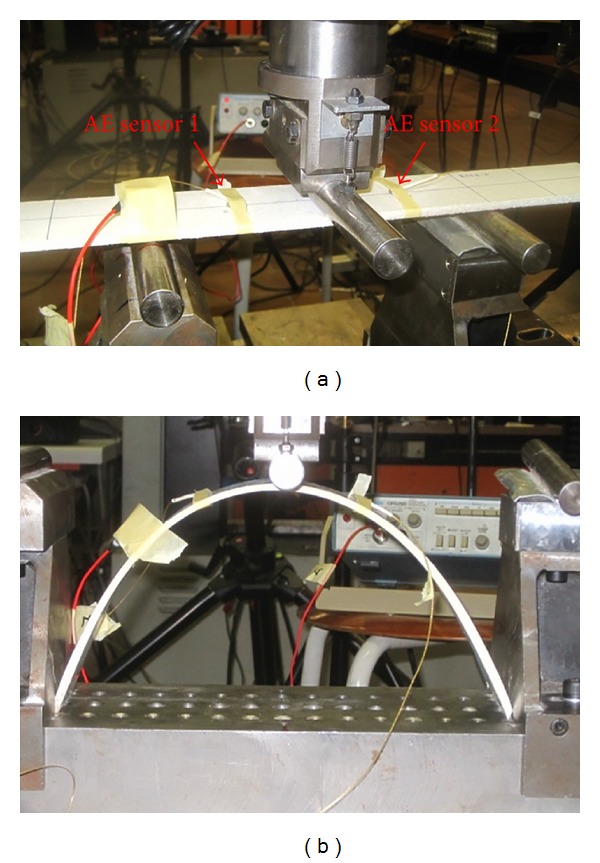
Three point bending set up for (a) straight beam, (b) arch beam.

The straight and curved specimens were loaded in a three-point bending test, as seen in Figure 2. The straight beam was simply supported. The span between the supports was 200 mm. The supports of the curved beam are pinned with a span of 282 mm. An INSTRON 5885H universal testing machine fitted with a load cell of 10 kN was used. The testing was displacement controlled, with a rate of 1 mm/min.

## 4. Tensile and Bending Behaviour

**Figure 3:**
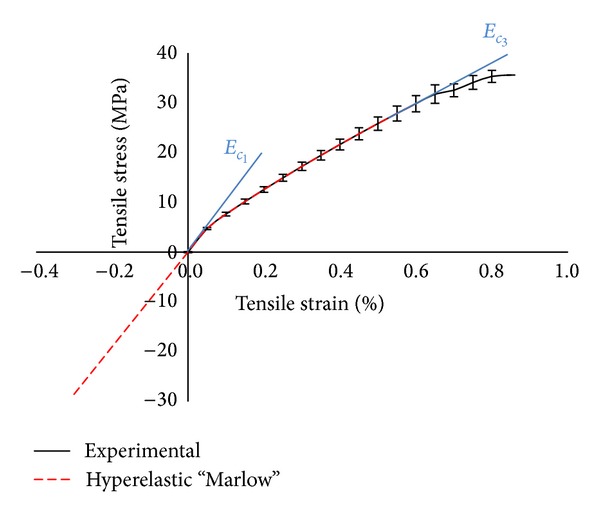
Stress-strain curves obtained from tensile tests on 8-layer TRC specimens.

The black continuous line in Figure 3 shows the average stress-strain curves obtained from 10 tested samples. The discontinuous red line represents the stress-strain curve computed with the calibrated hyperelastic “Marlow” model in Abaqus [11].

**Figure 4:**
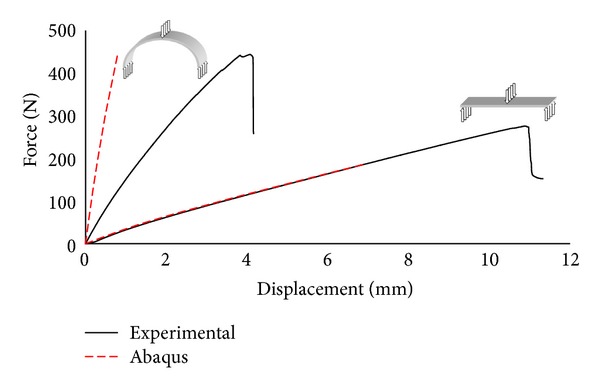
Force-deflection curves obtained from 3-point bending (curved and straight beams) and FEM simulation (solid lines stand for experimental curves, dashed lines for numerical).

Concerning bending, the force-deflection curves obtained from the 3-point bending on a straight and curved beam are shown in Figure 4. The red discontinuous line represents the force deflection curves obtained from the Abaqus bending model while the solid lines stand for experimental results.

**Figure 5:**
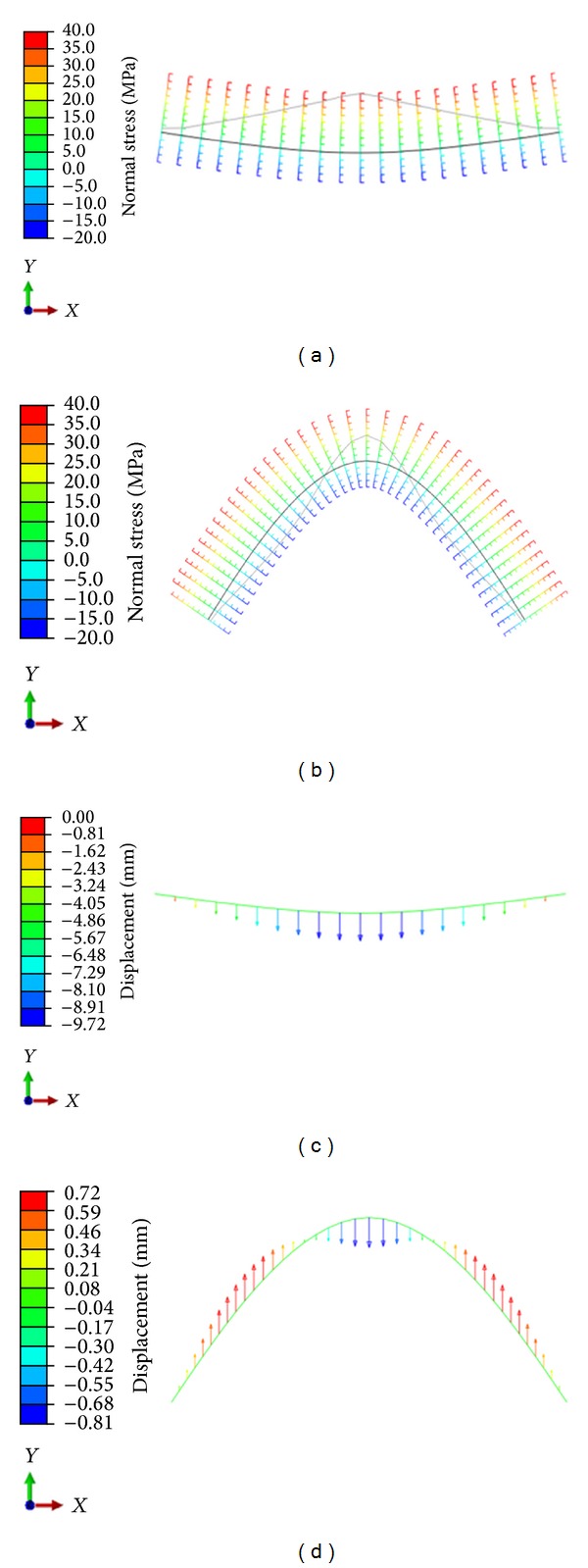
Normal stress (MPa) on the longitudinal axis of (a) straight and (b) curved beams. Vertical displacement of (c) straight and (d) curved beams.

The agreement between the numerical and experimental results is excellent for the thin straight beam in bending even with a model which is not taking in account shear [12]. However, there is no agreement for the arch shaped specimen. This implies that interlaminar shear starts playing an important role and not taking it into account results in strong discrepancies between the predicted and the actual behaviour [13]. Figures 5(a) and 5(b) show the normal stress, at the bottom of the section, along the longitudinal axis of the straight and the curved beams, respectively. It is obvious that the straight beam in Figure 5(a) is characterized by positive stress (tension) at the bottom of the section over the entire length of the specimen. However, in a curved beam the load will be transversal to the textile reinforcement layers only in the midsection. When increasing the curvature the forces will become more in line with the reinforcement layers. Curving a beam will result in the shift of bending (force perpendicular to reinforcement) to compression-shear (force aligned with reinforcement), as has been seen in [13]. Figures 5(c) and 5(d) depict the vertical displacement. While the straight beam moves downwards for its whole length, the displacement is more complicated for the curved one. Near the midspan the displacement is downward while at the sides it becomes positive (upward) due to the change of shape of the arch which tends to “open” under the vertical load. These results will be discussed along with the AE monitoring that follows.

## 5. Acoustic Emission Monitoring

For the specific experiment, two miniature AE sensors of the “pico” type (mistrasgroup) were used. They exhibit a broadband response and peak sensitivity at 450 kHz. They were attached by means of tape on the side surface of the components. A layer of Vaseline was used between the sensors and the specimen's surface for acoustic coupling. The separation distance of the sensors was 100 mm, as they were placed 50 mm in either side of the centre load application point (see Figure 2). The signals that exceeded 35 dB were preamplified by 40 dB and acquired in a PAC micro-II 8-channel board. AE monitoring was active during the whole loading pattern of each specimen, while activities with “zero” energy were not recorded. The larger transducer seen on the left of the photographs of Figure 2 was for exciting signals for ultrasonic monitoring which is not presented herein. In order to increase the reliability of the analysis and exclude possible noises the information of the AE “events” is only used. AE event is the source of the emission and is connected to a crack propagation incident. In the specific case one event leads to acquisition of one “hit” on each of the sensors (two hits in total) in a short time window. By knowing the pulse velocity of the medium and the acquisition time on each sensor, linear location of the sources along the axis of the specimen is automatically conducted. The pulse velocity of the medium was measured along the longitudinal axis by pencil lead breaks before the experiment and was found equal to 2700 m/s in average.

## 6. AE Results and Discussion

**Figure 6:**
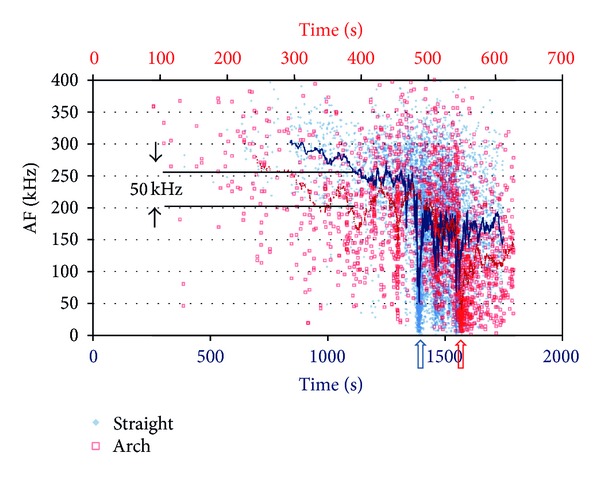
Average frequency (AF) for the straight and the curved plate (arch). The lines are the moving average of the recent 30 points.

In this study we will focus on the two extremes, namely, the straight and the arch with the maximum curvature, which are also shown in Figures 2(a) and 2(b).  Figure 6 shows the AF history for the AE hits for both the straight and curved (arch) plates. Each dot is the AF of one single AE signal while the time of acquisition is shown on the horizontal axis. A strong overlap is exhibited for the two populations straight and arch. The AF for the straight beam starts at an average of 300 kHz and smoothly decreases to approximately 220 kHz up to the moment of load drop (shown by an arrow on the horizontal axis). Until that moment matrix crack is reasonably active while after the serious cracking events that occur when the load bearing capacity of the beam is reached, fiber pull out and debonding between the successive layers are activated. This is why the AF drops to much lower values (moving average down to 50 kHz). The behaviour of the curved beam (arch) is similar but with distinct differences. Up to the fracture moment the AF decreases smoothly but it is constantly lower than the straight beam's AF. It starts at approximately 270 kHz and reaches 170 kHz just before load peak. This difference in average value of approximately 50 kHz indicates that the fracture mechanism in the arch includes more shear characteristics compared to the pure bending of the beam. Consequently, it clearly decreases at the moment of load drop indicating the activation of excessive debonding events. After the load drop points, the behaviour does not change much for the two beams since fracture of both is dominated by pull out and delaminations. However, the distinct differences in the first stage show that the shape of the beams actually influences the stress field and the dominant fracture mechanisms. The curved beam undertakes much more shear which is exhibited by lower frequency content of acoustic emissions. This confirms the conclusion of the modeling according to which for a straight beam neglecting the interlaminar shear is not crucial, while it becomes important for the modeling of the curved beam.

**Figure 7:**
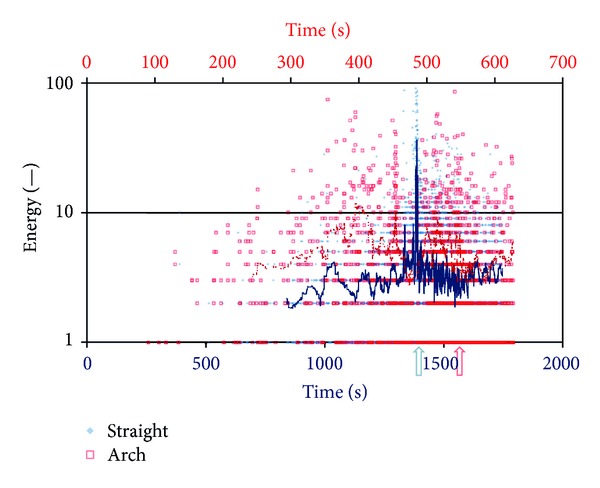
AE energy for the straight and the curved plate (arch). The lines are the moving average of the recent 30 points.

Focusing on the emitted energy for the two beams similar trends are noticed; see Figure 7. After the moments of load peak the behaviour is quite similar. However, at earlier stages of loading the energy emitted by a typical event of the arch is certainly higher (typically more than double) than the straight beam. This indicates that the events related to shear stresses are of higher intensity, compared to the pure matrix cracks that are characteristic of the straight beam.

In order to discuss on a more standard basis, the average values of these AE parameters along with AE durations are shown in Table 2. The AE activity of arch beams before the load peak is characterized by lower frequency, longer duration, and higher energy. After the peak the differences are much smaller; the average frequency is around 150 kHz for both specimens while the other parameters also seem to converge between the straight and arch geometries. This is due to the common damage mechanism of pull out and delaminations that is dominant after the matrix is fully cracked. Before that moment the data suggest that the straight beam sustains more normal stresses due to pure bending (short signals of higher frequency), while the curved undertakes stronger shear stresses due to the arch geometry. These significant changes before and after the maximum load have been observed previously in several fiber reinforced materials like ceramics [14], steel fiber reinforced concrete [15], and laminated composites [16]. Mainly it is the manifestation of fiber pull out and debonding, the shearing nature of which creates emissions with long duration and low frequency.

**Figure 8:**
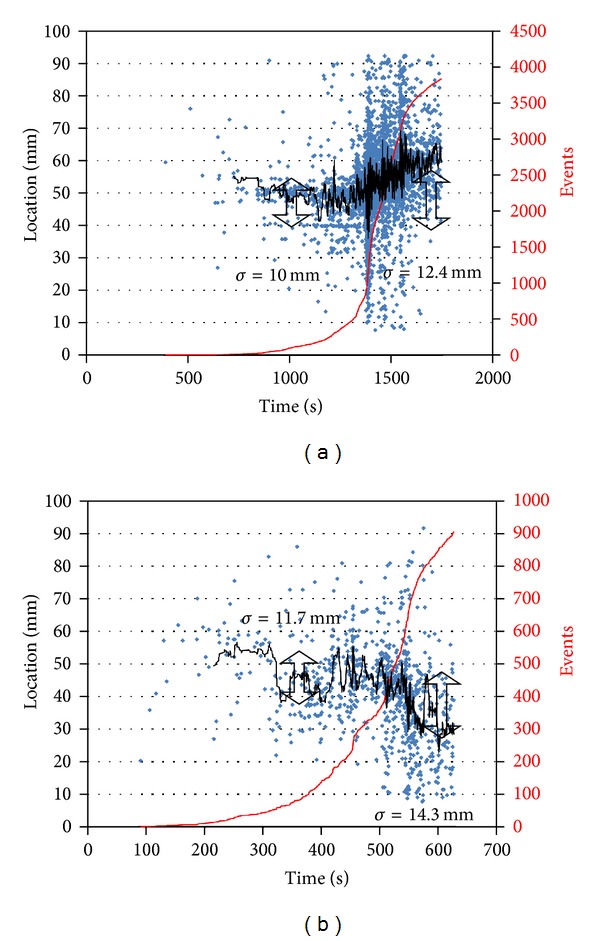
Location and cumulative number of AE events during loading of (a) the straight beam and (b) the arch beam.

As already mentioned AE source location took place and the analysed data population belongs to the classified events in order to increase the reliability.  Figure 8(a) shows the location results for the straight beam along with the cumulative number of events. Each dot is the calculated distance of the fracture source events from the first sensor (distance between the sensors is 100 mm). The slight black line is the moving average of the recent 30 points for clarity. The events initially are located very close to the centre of the beam as should be expected in three-point bending. There is a standard deviation, *σ*, of 10 mm around the mean value at the early stages of loading. However, later after the major fracture moment and load drop the mean value seems to shift away from the centre and the standard deviation considerably increases. This shows that although damage initiates at the centre due to the three-point bending setup, after the load drop, damage has the tendency to shift to one of the edges while the damaged area becomes wider (*σ* = 12.4 mm).

Results concerning the arch geometry show again small but characteristic differences (Figure 8(b)). Although the moving average line of AE location is close to the centre, the values exhibit a higher standard deviation (11.7 mm compared to 10 mm for the straight beam). After the load drop, again the mean value seems to shift to one of the sides while the widespread of the emission as seen by the standard deviation is increased to 14.3 mm. Therefore, it is a common place that the width of the fracturing zone increases as delaminations become more frequent at the end. This is reasonable since initially the three-point bending dictates the start of cracking at the centre due to maximum bending moment while once the delaminations start to occur, the damage becomes more widespread to both directions.

In Figure 8 the cumulative number of events is also seen for both cases. The straight beam exhibited a much higher activity in terms of the population of events. This implies that matrix cracking occurs with more events but of smaller intensity compared to the case of an arch beam where the total number of events is one-fourth but the average intensity is more than double.

The above discussion shows that simple AE descriptors can be used to distinguish the different fracture stages in complex material like fiber reinforced cement. During loading, a succession of failure modes is usually exhibited: initially matrix cracking due to tensile loading and consequently shearing due to incidents like fiber pull out or debonding. AE can readily capture the differences and hence shed light into the ongoing fracture process in real time. Concerning the curved beam, the existence of shear stresses and shear-related damage is seen from the start of loading based on the performance of frequency and other AE waveform indicators. It is thus confirmed that, for the highly curved beam, modeling cannot be conducted based on normal stresses alone, since strong shearing is also active. One point that should be kept in mind in such analyses is related to the heterogeneity and shape of the monitored specimen/structure. In the specific case, the plate geometry induces plate wave dispersion, while the heterogeneous nature of the material induces strong scattering in addition to damping. Therefore, the elastic waveform is continuously changing from the point of the emission to the receiver position. Frequency characteristics are bound to decrease, while the waveform loses amplitude. Therefore, any specific AE value reported herein holds strictly for the specific test setup and sensor separation distance. In different cases (geometry, sensor type, and separation distance) similar trends are expected but the absolute values of the AE descriptors will not necessarily be the same.

## 7. Conclusions

The present study discusses the acoustic emission behaviour of TRC laminates under bending. The following important conclusions are drawn.

1. The acoustic emission activity changes during the fracture process indicating the shift from matrix cracking to debonding and fiber pull-out events. Specifically, AE frequency decreases while the duration of the waveforms increases after the major fracture that is accompanied by a clear load drop.
2. The change in the developed stress field induced by the curvature of the beams is confirmed by the AE behaviour. AE waveforms of the arch shaped beam exhibit more shear characteristics than the straight beam, namely, longer duration and lower frequency, even at early loading levels.
3. Fracture in the curved beams which include shear components comprises of fewer and stronger events.
4. AE study can supply valuable feedback to the numerical modeling in terms of the load at onset of fracture as well as the dominant mode of fracture, which cannot be assessed in real time by another conventional technique.
5. The bending behaviour of thin straight TRC beam with a IPC matrix can be predicted by using model in a FEM program “Abaqus.” Nevertheless, the choice and calibration of the material model “Marlow” will influence the prediction. Introducing curvature will introduce shear driven failure mechanisms. When neglecting this phenomenon in the FEM model strong discrepancies between the predicted and the actual behaviour will occur.

## Figures and Tables

**Table 1:** Dimensions of straight and curved specimens tested in bending.

	Thickness (mm)	Width (mm)	Support span (mm)	Fibre volume fraction *V*_*f*_ (%)	Number of layers
Straight	5.70	53.53	200.00	16.94	8
Curved	5.05	55.28	282.00	19.35	8

**Table 2:** Average values of AE descriptors for different loading stages of the beams.

	Av. freq. (kHz)	Energy (—)	Duration (*μ*s)
Straight beam			
Prepeak	258	2.8	224
Postpeak	154	4.4	616
Arch beam			
Prepeak	215	6.0	383
Postpeak	148	5.0	560
